# Informing disease modelling with brain-relevant functional genomic annotations

**DOI:** 10.1093/brain/awz295

**Published:** 2019-10-11

**Authors:** Regina H Reynolds, John Hardy, Mina Ryten, Sarah A Gagliano Taliun

**Affiliations:** 1 Department of Neurodegenerative Disease, University College London (UCL) Institute of Neurology, London, UK; 2 UK Dementia Research Institute at University College London (UCL), London, UK; 3 Center for Statistical Genetics and Department of Biostatistics, University of Michigan, Ann Arbor, Michigan, USA

**Keywords:** functional genomic annotations, cellular resolution, genome-wide association, neuropsychiatric disorders, neurodegenerative disorders

## Abstract

The past decade has seen a surge in the number of disease/trait-associated variants, largely because of the union of studies to share genetic data and the availability of electronic health records from large cohorts for research use. Variant discovery for neurological and neuropsychiatric genome-wide association studies, including schizophrenia, Parkinson’s disease and Alzheimer’s disease, has greatly benefitted; however, the translation of these genetic association results to interpretable biological mechanisms and models is lagging. Interpreting disease-associated variants requires knowledge of gene regulatory mechanisms and computational tools that permit integration of this knowledge with genome-wide association study results. Here, we summarize key conceptual advances in the generation of brain-relevant functional genomic annotations and amongst tools that allow integration of these annotations with association summary statistics, which together provide a new and exciting opportunity to identify disease-relevant genes, pathways and cell types *in silico*. We discuss the opportunities and challenges associated with these developments and conclude with our perspective on future advances in annotation generation, tool development and the union of the two.

## Teaser

How can we best translate the success of genome-wide association studies for neurological and neuropsychiatric diseases into therapeutic targets? Reynolds *et al*. critically assess existing brain-relevant functional genomic annotations and the tools available for integrating such annotations with summary-level genetic association data.

## Introduction

A better understanding of the genetic architecture of complex diseases/traits has the potential to improve our understanding of their pathophysiology. Indeed, identifying disease-relevant biological pathways has been the principal motivation for genome-wide association studies (GWASs). The assumption is that enhanced knowledge of these pathways will enable the translation of *in silico* discoveries to wet lab experiments and ultimately to the development of novel therapies and personalized treatments (Visscher *et al.*, 2017).

The past decade has seen a surge in the number of disease/trait-associated variants, with 71 673 variant-trait associations reported in the September 2018 update of the NHGRI-EBI GWAS catalogue (Buniello *et al.*, 2019). Key to this surge has been the union of studies to share genetic data as well as the availability of electronic health records from large cohorts for research use, which has increased sample sizes and, thus, statistical power to detect variant-trait associations (Pardiñas *et al.*, 2018; Wray *et al.*, 2018; Jansen *et al.*, 2019; Nalls *et al.*, 2019). For instance, the 2013 meta-analysis of Alzheimer’s disease analysed 17 008 cases and 37 154 controls, whereas the 2019 meta-analysis analysed 71 880 cases and 383 378 controls, an increase that was achieved using new techniques that incorporated family history from electronic health records and self-reported assessments from the UK Biobank into the study design (Lambert *et al.*, 2013; Jansen *et al.*, 2019). As a result of this increase, the number of near-independent loci associated with Alzheimer’s disease rose from 19 to 29.

While the rate of GWAS discovery has increased, our ability to interpret the biology of these associations has lagged behind considerably (Price *et al.*, 2015; Visscher *et al.*, 2017). Given that most risk loci are thought to operate by regulating gene expression, interpreting disease/trait-associated variants is reliant on (i) knowledge of (and means for assaying) gene regulatory mechanisms; and (ii) tools that permit integration of this knowledge with GWAS results. With advances in cell isolation and sequence-based -omic technologies, researchers can now assay the molecular phenotypes of gene regulation in an increasingly precise and granular manner. This has resulted in a surge in the generation of tissue- and cell-type-specific ‘functional genomic annotations’, a term used to denote regions of the genome to which functional information (e.g. DNA methylation, chromatin accessibility, or gene expression) has been attached (Pevsner, 2015; Gagliano, 2017). In parallel with this surge, an increasing number of computational tools have recently emerged that permit integration of functional genomic annotations with GWAS results, in particular, GWAS summary statistics (Pasaniuc and Price, 2017).

**Figure 1:**
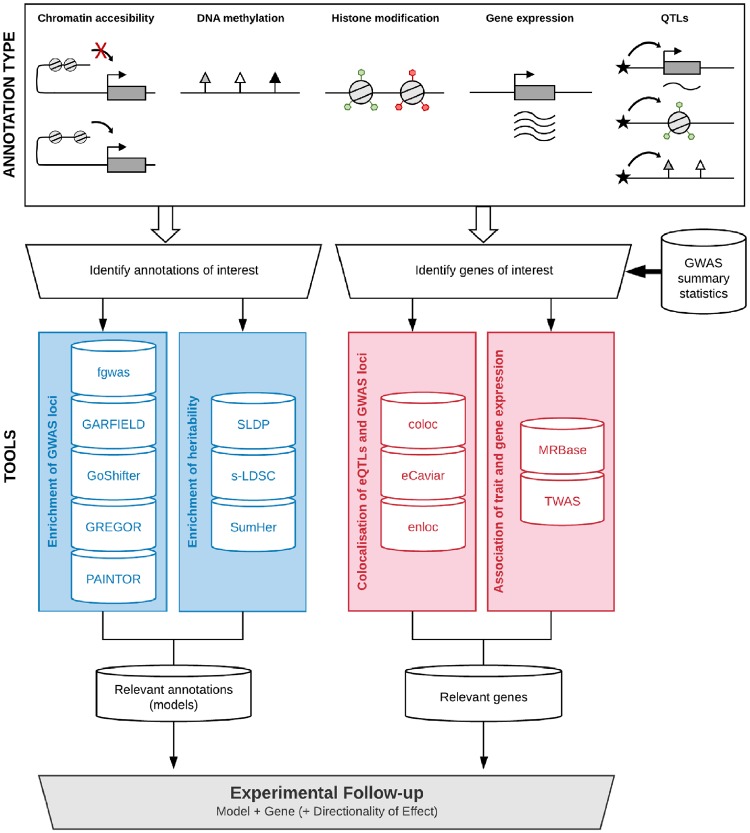
**Identifying annotations and genes of interest using GWAS summary statistics, relevant functional genomic annotations and genetic tools.** To follow up on GWAS risk loci experimentally, the wet lab researcher requires disease-relevant model systems, biological pathways and genes, and ideally some indication of how disease affects gene expression or regulation (i.e. directionality of effect). Identifying annotations of interest and genes of interest are complementary approaches that combine to constrain the model systems, gene targets and gene-specific pathways to pursue in functional experiments. For a description of the various functional genomic annotation types and an overview of the tools see Box 1 and Table 2, respectively.

With these advances, the mismatch between the rate of discovery versus that of interpretation appears set to change. In particular, these advances provide a new opportunity to identify the relevant cell types for GWAS risk variants *in silico*, in addition to genes and biological pathways of interest, as demonstrated for schizophrenia and Parkinson’s disease (Skene *et al.*, 2018; Bryois *et al.*, 2019; Reynolds *et al.*, 2019). This knowledge can inform our understanding of disease aetiology and experimental modelling, constraining the number of potential experimental models to a testable few (Fig. 1).

In this review, we highlight key conceptual developments in the generation of functional genomic annotations and tools that integrate these annotations with GWAS results. We review the opportunities and challenges associated with these developments, enabling researchers to critically assess the interpretative value of an annotation when combined with GWAS and determine the appropriate tool for integration. Finally, we conclude with a discussion of where we expect the next major advances in annotation generation, tool development, and the union of the two to emerge.

## Functional genomic annotations

Functional genomic annotations are vital to the interpretation of GWAS-identified human complex trait/disease-associated variants, 88% of which are found in non-coding regions of the genome (Hindorff *et al.*, 2009). Indeed, trait/disease-associated non-coding variants have been found enriched in several functional annotations, including expression quantitative trait loci (eQTLs) and measures of chromatin accessibility, such as deoxyribonuclease I (DNase I) hypersensitive sites (DHSs) and transposome hypersensitive sites (THSs) (Nicolae *et al.*, 2010; Grundberg *et al.*, 2012; Maurano *et al.*, 2012; Lake *et al.*, 2018). These enrichments are often more prominent in tissues or cell types with biological relevance to the associated trait/disease, thus motivating the exploration of annotations with higher tissue and cellular resolution (Grundberg *et al.*, 2012; Maurano *et al.*, 2012; Raj *et al.*, 2014; Finucane *et al.*, 2018; Lake *et al.*, 2018; Skene *et al.*, 2018).

**Figure 2:**
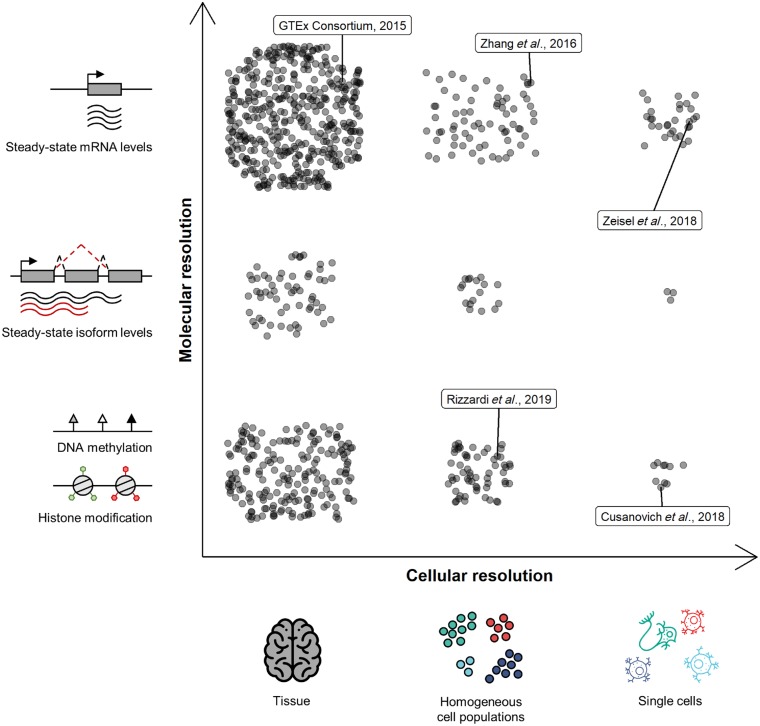
**The 2D space of cellular and molecular resolution.** Individual functional genomic annotations can be thought to lie somewhere on the axes of cellular resolution (spanning from whole tissue to single cells) and molecular resolution (spanning across epigenetic phenotypes, transcriptomic phenotypes and intermediates between the two). Points on the plot are purely illustrative, roughly depicting the relative number of functional annotations in each discrete population, with the most annotations currently found in the category of tissue-level steady-state mRNA levels and the least in the category of single cell steady-state isoform levels. To illustrate this categorization, examples of functional annotations highlighted in this review have been labelled. We expect that with future developments, the axis of molecular resolution will become increasingly populated with intermediate phenotypes such as steady-state isoform levels and various other RNA processing steps. Thus, in the future, populations within a category of cellular resolution will become less discrete across molecular phenotypes. For a description of above-mentioned molecular phenotypes and how they are assayed, see Box 1, and for further details on labelled functional annotations, see Table 1. Brain and single cell icons made by Eucalyp, Freepik and Smashicons from www.flaticon.com.

In many respects, technological advances in annotation generation have been driven by the push for cellular resolution, which today spans from whole tissue to single cells. Concomitant with this advance has been the development of technologies that allow genome-wide assaying across molecular phenotypes, ranging from epigenomic modifications to transcriptomic gene expression profiling (Box 1 and Fig. 2). While many of the modalities used to assay these molecular phenotypes were originally developed for whole tissue samples, the past few years has seen these scaled for use with single cells too. Thus, most functional genomic annotations can be thought to lie somewhere in the 2D space created by these two axes of information (Fig. 2). The following section highlights the progress and ongoing evolution of annotation generation within the axis of cellular resolution in the context of the human brain (see Table 1 for summary of referenced annotations).

Box 1 Assaying the epigenome and the transcriptome

**Assaying the epigenome**

The epigenome is used to describe the genome-wide collection of sequence-independent processes that modulate gene expression patterns. This modulation is typically enacted by altering chromatin, the protein-DNA complex in which genetic information is packaged. Thus, the epigenome covers several layers of information, including: epigenetic modifiers (DNA methylation and covalent modification of histones), chromatin accessibility (profiling of open and closed chromatin, which reflect active and inactive DNA regulatory elements, respectively), and higher-order chromatin architecture (Rivera and Ren, 2013; Allis and Jenuwein, 2016; Klemm *et al.*, 2019).

In eukaryotes, DNA methylation occurs most commonly at cytosine residues (specifically on their fifth carbon, hence the abbreviation 5mC). Derivatives of 5mC, including 5-hydroxymethylcytosine (5hmC), also exist. These are generated as intermediates in the demethylation of 5mC to cytosine (Schübeler, 2015). While several methods exist to assay DNA methylation, the chemical conversion method otherwise known as bisulphite-sequencing (BS-seq) has long been considered the gold standard (Rivera and Ren, 2013; Schübeler, 2015; Kelsey *et al.*, 2017). In this method, bisulphite conversion of unmethylated cytosines is coupled with whole-genome sequencing. Importantly, 5mC and 5hmC are indistinguishable in BS-seq, and it is only with the development of oxidative BS-seq (oxBS-seq) that researchers have begun to accurately distinguish between individual 5mC and 5hmC sites (Booth *et al.*, 2012).

 Histones constitute the proteins around which DNA is wrapped to form nucleosomes. They are subject to several post-translational modifications at specific amino acids that have been associated with both open and closed chromatin states (depending on the mark and the histone context surrounding it). Histone modifications can be mapped by chromatin immunoprecipitation, wherein DNA-protein complexes containing a protein of interest are immunoprecipitated, followed by sequencing in a method known as chromatin immunoprecipitation followed by sequencing (ChIP-seq) (Rivera and Ren, 2013).

Chromatin accessibility can be thought to exist on a continuum from inaccessible to accessible. For simplicity, this is typically reduced to two states, closed and open, and occasionally a third permissive state, which describes chromatin that is sufficiently dynamic to establish an open conformation. As with DNA methylation, several techniques exist to quantify chromatin accessibility. Two of the most commonly used are DNase I hypersensitive site sequencing (DNase-seq) and assay for transposase-accessible chromatin using sequencing (ATAC-seq). DNase-seq uses the endonuclease, DNaseI, to cleave out DNA in accessible chromatin, which is then sequenced. ATAC-seq, on the other hand, uses a mutated Tn5 transposase, which simultaneously cleaves and ligates sequencing adaptors into regions of open chromatin, which are thereafter PCR amplified and sequenced (Klemm *et al.*, 2019). Chromatin structure can also be considered in terms of its short- and long-range interactions, which can be both activating and repressive, and is assayed in an unbiased, global manner using genome-wide chromosome conformation capture (Hi-C) (Schmitt *et al.*, 2016).

For detailed reviews of the various technologies available for assaying the epigenome of bulk- and single-cell populations please refer to (Rivera and Ren, 2013) and (Kelsey *et al.*, 2017), respectively.

**Assaying the transcriptome**

Gene expression (often used synonymously with RNA expression) denotes the process of generating a functional product from a gene. In this review, gene expression is exclusively used to indicate expression of a gene at the transcriptomic level (i.e. RNA abundance). Early genome-wide measurements of gene expression were performed with a hybridization-based microarray method, wherein fluorescent cDNA (generated from RNA) is hybridized to DNA probes and relative cDNA abundance measured by fluorescence. RNA-sequencing, which uses next-generation sequencing to quantify RNA species, is currently the dominant technology for transcriptomic profiling because of its unbiased nature and broad dynamic range (Mortazavi *et al.*, 2008; Wang *et al.*, 2009; Zhao *et al.*, 2014). This dominance is reflected in its use in a wide variety of methods, which allow quantification of various RNA processing steps from transcription to translation, as well as its use in single-cell measurements (Kolodziejczyk *et al.*, 2015; Nussbacher *et al.*, 2015). However, current measurements of the transcriptome only capture a static snapshot in time, which reflects the combined output of RNA synthesis, splicing and degradation. While consideration of the ratio of unspliced RNA to spliced RNA can be used to infer expression dynamics, these methods remain probabilistic in nature (Gray *et al.*, 2014; Gaidatzis *et al.*, 2015; La Manno *et al.*, 2018).

**Quantitative trait loci**

Quantitative trait loci (QTLs) refer to regions of the genome wherein DNA variation is statistically associated with variation in a quantitative phenotypic trait. In principle, phenotypes include any form of quantitative trait, with classical examples being height and weight (Abiola *et al.*, 2003). With the advent of genome-wide transcriptomic profiling, it was suggested that traditional QTL analyses be extended to include variation of gene expression (eQTLs) (Jansen and Nap, 2001). QTL analyses have now been extended to include many molecular phenotypes, such as: RNA splicing (sQTLs), chromatin accessibility (caQTLs), DNA methylation (mQTLs), histone modification (hQTLs) and even cell-type proportions (fQTLs) (Albert and Kruglyak, 2015; Ng *et al.*, 2017; Wang *et al.*, 2018).

**Table 1:** Highlighted brain-relevant functional genomic annotations

Cellular resolution	Molecular phenotype	Web resource	Reference	Species
**Tissue**	Chromatin accessibility	BOCA: http://icahn.mssm.edu/boca	Fullard *et al.*, 2018	Human
	Chromatin accessibility, gene expression	CommonMind: http://commonmind.org/WP	Fromer *et al.*, 2016	Human
	Gene expression, eQTLs	BrainSeq Consortium: http://eqtl.brainseq.org/	BrainSeq: A Human Brain Genomics Consortium, 2015	Human
		GTEx: http://www.gtexportal.org/home/	GTEx Consortium, 2015	Human
		UKBEC: http://braineac.org/	Ramasamy *et al.*, 2014	Human
	Gene expression, *in situ* hybridization, MRI	Allen Brain Atlas: http://human.brain-map.org/	Hawrylycz *et al.*, 2012, 2015	Human
	Chromatin accessibility, epigenetic modifiers, gene expression, QTLs	AMP-AD Knowledge Portal: https://www.synapse.org/#!Synapse:syn2580853/wiki/	https://www.nia.nih.gov/research/amp-ad	Mouse, *Drosophila*, human
		Brain xQTL Serve: http://mostafavilab.stat.ubc.ca/xQTLServe/ [Uses ROSMAP samples]	Ng *et al.*, 2017	Human
		FANTOM5: http://fantom.gsc.riken.jp/5/		Mouse, human
		PsychENCODE: http://psychencode.org/	PsychENCODE Consortium, 2018	Human
		PsychENCODE Knowledge Portal: https://www.synapse. org/#!Synapse:syn4921369		
		PEC Capstone Collection: http://resource.psychencode. org/# and https://www.synapse.org/#!Synapse:syn120 80241		
		ROSMAP: https://www.synapse.org/#!Synapse:syn3219045	De Jager *et al.*, 2018*a*	Human
**Cell type**				
Homogeneous cell populations	DNA methylation	No web resource. Data available from Gene Expression Omnibus under GSE96615. Processed data also available through UCSC hub: http://genome.ucsc.edu/cgi-bin/hgTracks?db = hg19&hubUrl = https://s3.us-east-2.amazonaws.com/brainepigenome/hub.txt.	Rizzardi *et al.*, 2019	Human
	Gene expression	http://www.brainrnaseq.org/	Zhang *et al.*, 2014, 2016	Mouse, human
		BRAINcode Project: http://www.humanbraincode.org/	Dong *et al.*, 2018	Human
Single-cell analyses	Chromatin accessibility	http://atlas.gs.washington.edu/mouse-atac/	Cusanovich *et al.*, 2018	Mouse
	Gene expression	The Broad Institute: https://portals.broadinstitute.org/single_cell		Mouse, Human
		https://portals.broadinstitute.org/single_cell/study/dronc-seq-single-nucleus-rna-seq-on-human-archived-brain	Habib *et al.*, 2017	Mouse, human
		http://dropviz.org/	Saunders *et al.*, 2018	Mouse
		http://mousebrain.org/	Zeisel *et al.*, 2018	Mouse
		Tabula Muris: https://figshare.com/projects/Tabula_Muris_Transcriptomic_characterization_of_20_organs_and_tissues_from_Mus_musculus_at_single_cell_resolution/27733	Habib *et al.*, 2017	Mouse, human
	Chromatin accessibility, gene expression	The BRAIN Initiative: https://www.braininitiative.nih.gov/	Koroshetz *et al.*, 2018	Human
		Human Cell Atlas: https://www.humancellatlas.org/	Regev *et al.*, 2017	Human
		PsychENCODE: http://psychencode.org/	PsychENCODE Consortium, 2018	Human
		http://brainome.org [Part of BRAIN Initiative]	Luo *et al.*, 2017	Human
		No web resource. Data available from Gene Expression Omnibus under GSE97942.	Lake *et al.*, 2018	Human

## Cellular heterogeneity: impact and resolution

The brain is a heterogeneous tissue organized into functionally separate but interconnected regions, an observation that is mirrored in regional transcriptomes and their regulation (Oldham *et al.*, 2008; Ramasamy *et al.*, 2014). In turn, brain regions reflect a diverse collection of underlying cell types, with each cell type often exhibiting their own specific regulatory features (Cuevas-Diaz Duran *et al.*, 2017). When averaging features across a tissue, specific features may be diluted or altogether masked, which at worst could lead to an incorrect interpretation (Trapnell, 2015). As an example, DNA methylomes were until recently thought to be largely homogeneous between brain regions, a conclusion based on bulk tissue analyses (Illingworth *et al.*, 2015). However, a study separating brain tissue into neuronal and non-neuronal populations by fluorescence-activated nuclear sorting (FANS) demonstrated that methylation differences amongst neurons from distinct brain regions were masked when combined with non-neurons, which showed fewer methylation differences across brain regions (Rizzardi *et al.*, 2019). In view of this conclusion and the observation that varying cell-type proportions largely account for cross-population variations in bulk tissue gene expression (Wang *et al.*, 2018), it is clear that cellular heterogeneity must be accounted for.

Several strategies, often determined by the technology of the time, have been used to tackle the challenge of providing molecular phenotypes at high cellular resolution. These approaches include the use of whole tissue combined with *in silico* methods, in addition to targeted isolation of homogeneous cell populations and ultimately, unbiased isolation of single cells.

### Whole tissue as a proxy for cell types

With the extension of eQTL studies to humans (Cheung *et al.*, 2005; Stranger *et al.*, 2007), the early 2010s saw a surge in the number of whole brain tissue resources. Prominent resources include: (i) the Allen Human Brain Atlas (Hawrylycz *et al.*, 2012); (ii) the Genotype-Tissue Expression (GTEx) consortium database (GTEx Consortium *et al.*, 2015); and (iii) the United Kingdom Brain Expression Consortium (UKBEC) database (Ramasamy *et al.*, 2014). While the first focused on anatomical comprehensiveness, performing microarray profiling of ∼900 anatomically defined sites in six individuals, the latter two favoured larger sample sizes, covering over 10 brain regions with independent sample sizes ranging from 67 to as many as 173 individuals.

These early studies sought to define ‘normal’ transcription and its regulation within the brain, and thus were characterized by their use of neuropathologically confirmed control individuals. On the other hand, later consortia-driven efforts have primarily been disease-focused often assuming a case-control structure, as in the case of the neuropsychiatric consortia PsychENCODE, CommonMind and BrainSeq (Akbarian *et al.*, 2015; BrainSeq: A Human Brain Genomics Consortium, 2015; Fromer *et al.*, 2016; PsychENCODE Consortium, 2018). As an exception, one of the largest resources for neurodegeneration, ROSMAP, combines two prospective studies of ageing (The Religious Order Study and the Memory and Ageing Project) (De Jager *et al.*, 2018*a*). As a result of its prospective nature, this resource has highlighted the common occurrence of neuropathology in older individuals with no cognitive impairment and the co-existence of multiple neuropathologies in individuals with a definitive Alzheimer’s disease diagnosis, emphasizing the need for additional population-based prospective studies (De Jager *et al.*, 2018*b*). Collectively these resources amongst others have been critical for the identification of transcriptomic and epigenomic alterations in disease states.

Common to all tissue-based resources is the use of *in silico* methods to either (i) mitigate the effects of cellular heterogeneity; or (ii) infer cell-type specific information. The first uses deconvolution approaches to estimate cell-type proportions, which can then be used to correct bulk data (Newman *et al.*, 2015; Teschendorff and Zheng, 2017; Wang *et al.*, 2018). The statistical algorithms underlying deconvolution come in a variety of flavours, broadly classified as reference-based (i.e. they require *a priori* knowledge of cell types and reference profiles) and reference-free. As with all algorithms there are associated disadvantages, with the performance of reference-based approaches reliant on the quality of reference profiles and the performance of reference-free algorithms dependent on valid model assumptions (for a full review see Teschendorff and Zheng, 2017). The second is most commonly achieved using weighted gene co-expression network analysis (WGCNA), which seeks to identify sets of genes, referred to as ‘modules’, with highly correlated expression across biological samples. Initially applied to investigate transcriptional organization of the brain, it was soon found that cellular heterogeneity constituted the major organizing principle of derived networks, with investigators able to identify and map modules to specific cell types (Oldham *et al.*, 2008; Miller *et al.*, 2010; Hawrylycz *et al.*, 2012). This approach has since been widely applied to both case-control and prospective cohorts in the fields of neuropsychiatry and neurodegeneration, with modules used as annotations to identify disease-relevant cell types, as with microglia and oligodendrocytes in Alzheimer’s disease (Seyfried *et al.*, 2017; Allen *et al.*, 2018) or neuroinflammatory astrocytes and microglia in autism spectrum disorder, bipolar disease and schizophrenia (Gandal *et al.*, 2018).

The key advantage of using such *in silico* methods is the preservation of tissue-level complexity with added cellular resolution; however, while deconvolution approaches exist for both epigenomic and transcriptomic data, network approaches are currently limited to transcriptomic data. Furthermore, cellular inferences rely upon prior knowledge of cell-type-specific expression.

### Targeting homogeneous cell populations

Parallel to these whole-tissue approaches has been the development of laboratory techniques allowing targeted isolation of homogeneous cell populations. Until the advent of high-throughput single-cell RNA-sequencing, which has proven to be a key turning point in the drive for increased cellular resolution, the primary focus was on traditional approaches, including laser capture microdissection (LCM), fluorescence-activated cell sorting (FACS), immunopanning and translating ribosome affinity purification (TRAP). These techniques continue to be used today, both as an alternative to and to complement single-cell studies.

LCM, originally introduced in 1996 (Emmert-Buck *et al.*, 1996), combines microscopic visualization with a laser beam (typically infrared or ultraviolet) to isolate targeted cell types from histological specimens and fresh or frozen tissue sections (Datta *et al.*, 2015; Valihrach *et al.*, 2018). Combined with microarray or RNA-sequencing, this has been used to analyse the transcriptome of distinct neuronal populations in numerous studies (Chung *et al.*, 2005; Simunovic *et al.*, 2009; Bandyopadhyay *et al.*, 2013; Dong *et al.*, 2018). Of note, is the recent BRAINcode project, a substantial undertaking wherein total RNA from ∼40 400 laser captured dopaminergic and pyramidal neurons from 99 human post-mortem brains was deeply sequenced, identifying ∼71 000 transcribed non-coding elements, many of which appear to represent active enhancer elements (Dong *et al.*, 2018). This study highlights the strengths of LCM, namely the ability to isolate morphologically-distinct cell types without the need for genetic labelling and the ability to deeply sequence cell types while preserving spatial information (Kuhn *et al.*, 2012; Zechel *et al.*, 2014; Dong *et al.*, 2018). However, LCM remains a slow and laborious technique, and suffers from substantial disadvantages including RNA degradation, loss of long neuronal processes, and contamination from other cells (Clément-Ziza *et al.*, 2008; Datta *et al.*, 2015; Krishnaswami *et al.*, 2016; Lake *et al.*, 2016; Valihrach *et al.*, 2018).

FACS, on the other hand, is high throughput and capable of capturing rare cell types with little contamination. Together with advances in recombineering and transgenesis, in particular the ability to generate transgenic mice expressing a fluorescent reporter gene (e.g. enhanced green fluorescent protein, EGFP) in specific cell types, this approach was key to several ground-breaking studies from the early 2000s (Arlotta *et al.*, 2005; Lobo *et al.*, 2006; Sugino *et al.*, 2006). The studies combined FACS with microarray analysis to identify neuronal subpopulations in the adult mouse brain, including corticospinal projections neurons (Arlotta *et al.*, 2005), GABAergic and glutamatergic neurons (Sugino *et al.*, 2006), and striatal projection neuron subtypes (Lobo *et al.*, 2006).

An equally important development in targeted cell-type isolation was immunopanning, which uses cell surface markers to purify specific cell types. Together with FACS, this was instrumental in comprehensive gene expression studies, wherein major cell types (astrocytes, endothelial cells, microglia, neurons, oligodendrocytes and oligodendrocyte precursor cells) were isolated from adult mouse or human brain and profiled using microarray or RNA-sequencing (Cahoy *et al.*, 2008; Zhang *et al.*, 2014, 2016). These studies have been especially informative in large part due to the user-friendly format in which these data were released (http://www.brainrnaseq.org/) (Zhang *et al.*, 2016).

FACS and its derivatives, such as FANS, remain widely used. Indeed, recent studies of the epigenome have primarily used FANS, because of the ease with which neurons can be distinguished from glial cell populations using the neuronal marker NeuN (Girdhar *et al.*, 2018; Rizzardi *et al.*, 2019). However, both immunopanning and FACS approaches rely upon physical enrichment of target cell populations from acutely dissociated cells prior to lysis and RNA extraction. This isolation process is lengthy and there is a risk of altering transcriptional profiles. As an alternative methodology, TRAP was established by Doyle *et al.* and Heimen *et al.* in 2008. TRAP involves expression of an EGFP-L10a ribosomal protein in BAC mouse lines (transgenic lines wherein a reporter gene is placed under the control of the regulatory sequences of an endogenous gene of interest), and immunoaffinity purification of EGFP-tagged ribosomes. Thus, bacTRAP allows isolation of cell-type-specific polysome-bound mRNAs from whole-tissue homogenates, without the need for cellular isolation (Doyle *et al.*, 2008; Heiman *et al.*, 2008). Since 2008, TRAP and its variants have been used to profile gene expression in many different cell types of the mouse CNS, and has been noted for its higher sensitivity in detecting low-expressed cell-type-specific genes (as compared to some single-cell RNA-sequencing datasets) and its preservation of cellular components (e.g. dendrites and axons) (Knight *et al.*, 2012; Schmidt *et al.*, 2012; Ekstrand *et al.*, 2014; Shrestha *et al.*, 2015; Nectow *et al.*, 2017). While the information yielded from these studies is undeniably valuable, bacTRAP is, however, inherently limited to model organisms and *in vitro* systems because of its reliance upon genetic engineering.

### Single-cell analyses

With the advent of single-cell analyses, assessment of cell-to-cell heterogeneity across the transcriptome and, more recently, the epigenome has become an exciting new possibility. In the first study to apply this technology to the adult human brain (frontal cortex, *n* = 8), Darmanis *et al.* (2015) used gene expression from 466 cells to identify all major cell types (i.e. neurons, astrocytes, microglia, oligodendrocytes, endothelial cells) and characterize neuronal subtypes. Since then, advances in single-cell transcriptome profiling, in particular the development of ultra-high-throughput droplet-based systems (such as, inDrop, Drop-seq and 10X Genomics Chromium), wherein single cells are encapsulated in nanolitre droplets containing barcoded beads, have permitted simultaneous profiling of thousands of cells at a reduced cost (Zhang *et al.*, 2018). Equally important has been the adaptation of these high-throughput methods for use with isolated single nuclei (Habib *et al.*, 2017). A major impediment to single-cell human brain analyses has been the requirement for a single-cell suspension to be prepared from fresh tissue, thus limiting users to surgical specimens, which for the brain are only available from individuals undergoing surgical treatment, such as for epilepsy or a tumour. Single-cell isolation from brain tissue requires a harsh dissociation protocol, which has been associated with transcriptional artefacts and is known to bias cell type proportions; that is, some cell types, especially neurons, are more vulnerable to dissociation (Habib *et al.*, 2016; Krishnaswami *et al.*, 2016; Lacar *et al.*, 2016). With single-nucleus RNA-sequencing, however, there is no requirement for protease digestion or heating. This method thus preserves the transcriptional profiles, whilst allowing the use of frozen, archival samples, which opens the door to use of samples from deceased individuals with various neurodegenerative or neuropsychiatric disorders (Habib *et al.*, 2017).

Most recently, single-cell profiling of the human brain was extended to the epigenome with single-cell methylomes and later single-cell chromatin accessibility, as assessed by transposome hypersensitive site sequencing (scTHS-seq, a variant of ATAC-seq, which combines *in vitro* transcription with an engineered Tn5 transposase to achieve higher sensitivity) (Luo *et al.*, 2017; Lake *et al.*, 2018). In the latter study, scTHS-seq together with single-nucleus Drop-seq (snDrop-seq) was applied to >60 000 single cells from the human adult visual cortex, frontal cortex and cerebellum (in nine individuals: three for scTHS-seq and six for snDrop-seq), allowing a combined analysis of the transcriptome and epigenome (Lake *et al.*, 2018). While such approaches will be key to identifying regulatory elements and processes that shape cell-type identity, their strength also lies in the ability to use associations between molecular layers to complete missing data (Lake *et al.*, 2018).

These high-throughput single-cell and -nucleus analyses have transformed the field, with publications rapidly emerging that implicate often underestimated cell types in disease pathophysiology. Indeed, from April to May 2019 alone, three single-cell studies of Alzheimer’s patient and control brains were released, two of which implicated oligodendrocytes (Del-Aguila *et al.*, 2019; Grubman *et al.*, 2019; Mathys *et al.*, 2019). These studies add to a rapidly expanding collection of single-cell resources that are complemented by research endeavours such as The BRAIN Initiative (Koroshetz *et al.*, 2018) and the Human Cell Atlas (HCA) (Regev *et al.*, 2017), which aim to map cell types in the brain and beyond. However, as with any emerging technology, single-cell and -nucleus resources derived using high-throughput droplet-based technologies come with limitations including: (i) sparser data; (ii) lower molecular resolution; and (iii) differential cell type loss due to cellular isolation protocols, which require consideration prior to use as annotations (for further details see Box 2) (Kelsey *et al.*, 2017; Svensson *et al.*, 2017*a*; Bakken *et al.*, 2018*a*; Lake *et al.*, 2018).

Box 2 Important considerations in the use of high-throughput single-cell and -nucleus data

**Sparser data**

Genome coverage is typically lower in high-throughput droplet-based single-cell/nucleus experiments than in bulk sequencing experiments. As an example, Lake *et al.* in their dual profiling of the transcriptome and chromatin accessibility of >60 000 nuclei from three brain regions detected a median of 928 unique transcripts and 719 genes per nucleus, while coverage of open chromatin yielded a median of 10 168 unique reads per cell and 45 million unique reads per sample (Lake *et al.*, 2018). By comparison, a median of between 20 000 and 32 000 gene transcripts was detected by GTEx across bulk RNA-sequencing of 53 different tissues (GTEx Consortium, 2015), while a median of 88 million unique reads per sample was detected in a study of bulk chromatin accessibility from the dorsolateral prefrontal cortex (Bryois *et al.*, 2018). Unsurprisingly, in a power analysis of single-cell RNA-sequencing experiments, bulk RNA-sequencing was found to be more accurate (as determined by how close estimated relative abundance levels were to known abundance levels of input molecules), although accuracy of single-cell RNA-sequencing was still found to be high (Svensson *et al.*, 2017*b*).

**Lower molecular resolution**

Provided a sufficiently large sample size (i.e. sequencing more cells), it is possible to overcome the problem of genome coverage to, as a minimum, resolve cell-type diversity. However, the same opportunity to resolve transcript isoforms as in bulk RNA-sequencing is not yet feasible using droplet-based techniques. While methods exist to construct libraries with full-length transcripts from post-mortem samples, these preclude an early barcoding step, which is essential for distinguishing individual cells/nuclei in droplet-based methodologies (Krishnaswami *et al.*, 2016; Ziegenhain *et al.*, 2017). That is, full-length library constructions are incompatible with the parallel nature of droplet-based sequencing technologies, and thus remain low throughput and expensive (Ziegenhain *et al.*, 2017).

**Differential loss of cell types**

Some cell types may be lost because of cellular isolation protocols. In their transcriptomic profiling of the mouse nervous system, Zeisel *et al.* (2018) note that interneurons were undersampled (potentially as a result of their fragility), while other studies have found cortical paravalbumin-expressing neurons selectively depleted in cell suspensions derived from primary cortical tissue (Zeisel *et al.*, 2015; Tasic *et al.*, 2016). Thus, single-cell analyses of whole tissue cannot be considered completely representative of the same tissue *in vivo*.

**Correlation profiles between single cells and nuclei**

An important discussion point in the world of single-cell transcriptomics is the extent to which profiling single nuclei is an appropriate substitute for profiling of whole cells. Several comparative studies have determined that the average expression profile of single nuclei is well-correlated with the average profile of single whole cells, provides a similar separation of cell types, and represents tissue-level RNA expression (Lacar *et al.*, 2016; Habib *et al.*, 2017; Lake *et al.*, 2017; Bakken *et al.*, 2018*b*). However, other comparative studies have found preferential enrichment of certain transcripts within nuclear and cytoplasmic compartments, suggesting that caution should be applied and validation of an approach is key (Habib *et al.*, 2017; Lake *et al.*, 2017; Abdelmoez *et al.*, 2018; Bakken *et al.*, 2018*b*; Skene *et al.*, 2018).

### Summary

Technological developments over the past decade have defined the trajectory of annotation generation within the axis of cellular resolution. Notably, international consortia have played a large role in this generation; understanding and mapping cellular heterogeneity is a massive endeavour that requires such collaborative efforts. Today, there is no shortage of annotations available and with annotations emerging at a rapid rate, often with large sample numbers and increasing cellular resolution, the quantity is only set to rise.

For researchers wishing to use these annotations to interpret the biology of trait/disease-associated variants, there comes an added responsibility to critically assess the value of an annotation to inform biology. Ideally, an annotation should provide high cellular resolution across several molecular phenotypes. Furthermore, it should be anatomically comprehensive (in brain this would equate to a comprehensive coverage of brain regions) and offer high-depth genome-wide coverage. Unsurprisingly, no current annotations satisfy all these criteria, because of the exceptionally expensive nature of such an endeavour. Thus, increasing cellular resolution, as with use of single-cell analyses, often comes with a choice between anatomical comprehensiveness or high-depth genome coverage. This results in annotations such as those produced by Saunders *et al.* (2018) and Zeisel *et al.* (2018), which provide an impressive anatomical comprehensiveness at single-cell resolution (covering 9 and 19 regions in the nervous system, respectively), but with low sequencing depth and limited coverage of isoform usage. Likewise, increasing cellular resolution whilst also covering multiple molecular phenotypes is not without a sacrifice, as exemplified by PsychENCODE who have epigenomic and transcriptomic data at the level of tissues and homogeneous cell populations, in addition to single-cell transcriptomic data, but have chosen to focus almost entirely on one brain region, the dorsolateral prefrontal cortex (see PsychENCODE knowledge portal, Table 1).

In view of the above, it is important for data analysts to use a variety of annotations to inform trait/disease biology, such that they might sufficiently cover the two-dimensional space created by the axes of cellular and molecular resolution (Fig. 2). Furthermore, it highlights the need to prioritize which annotations should be generated next, a task that requires assessment of the value specific annotations add to GWAS interpretation, unbiased tools for this assessment, and a dynamic flow of information between annotation generators and their tool users.

## Tools for integrating functional genomic annotations with GWAS summary statistics

This naturally leads to the evaluation of computational tools that integrate functional genomic annotations with GWAS results. Collectively, these tools are of key importance in prioritizing annotations and genes of interest, and thus have the potential to both improve our understanding of the regulatory mechanisms driving disease-association signals and provide targets for downstream functional experiments. As would be expected, these tools have evolved as our understanding of biology is refined and as annotation throughput increases. To depict this evolution, we highlight key conceptual advances in the development of tools that integrate genomic annotations with GWAS and provide select tools to illustrate. Specifically, we highlight the following advances: a move from modelling a single ‘causal’ variant to modelling multiple variants; a shift from assessing genome-wide significant or sub-threshold single nucleotide polymorphisms (SNPs) to assessing SNPs genome-wide; the emergence of tools that infer unmeasured data; and finally, ease of use. When possible, we also provide disease-related examples in which selected tools have been successfully applied (Table 3).

**Table 3:** Selection of disease-related examples of successful application of tools for integration of functional genomic annotations with GWAS summary statistics

Tool	Disease/Trait	Application and results	Reference
**Identifying annotations of interest**			
fastPAINTOR	Prostate cancer	Using PAINTOR together with 20 functional categories previously implicated in prostate cancer, a significant enrichment of prostate cancer-associated variants was found in FOX1A-binding sites assayed in the LNCaP cell line (derived from androgen-sensitive human prostate adenocarcinoma cells) and at binding sites for the androgen receptor.	Mancuso *et al.*, 2016
	High density lipoprotein / low density lipoprotein / total triglycerides	Using fastPAINTOR targeted at putative pleiotropic regions within the three traits, liver H3K4me1 and H3K27ac were found to have a strong enrichment of ‘causal’ variants shared across all three traits.	Kichaev *et al.*, 2017
GARFIELD	Schizophrenia	GARFIELD was applied to 29 diseases/complex traits and to several annotations, including DHSs and histone modifications. Statistically significant enrichments were found for most traits considered, and highlighted clear differences in enrichment patterns amongst traits. Of note to the focus of this review, schizophrenia-associated variants were found enriched in DHSs from blood and foetal brain, and H3K27ac and H3K4me3 predominantly in tissues of the CNS and blood/immune tissue.	Iotchkova *et al.*, 2019
GoShifter	Rheumatoid arthritis	Enrichment of rheumatoid arthritis-associated loci was found in summit regions of H3K4me3 peaks (active enhancer) in CD4+ T-memory cells, even when jointly analysed with an aggregate of 118 different cell types and tissues (which included >10 other immune cells), while no enrichment was found in the aggregate 118 cell types when conditioned upon CD4+ T-memory cells.	Trynka *et al.*, 2015
GREGOR	Atrial fibrillation	Atrial fibrillation-associated variants were found to be strongly associated with varying active enhancers in adult and foetal heart tissue e.g. H3K27ac in adult right atrium and left ventricle, and H3K4me1 in foetal heart tissue, highlighting the importance of these loci in transcriptional regulation of the adult heart and development of the foetal heart.	Nielsen *et al.*, 2018
	Type 2 diabetes	Among 184 trait- and disease-associated SNP sets (downloaded from the NHGRI-EBI GWAS Catalogue), the only disease found to be significantly enriched in chromatin accessibility QTLs from human pancreatic islet cells was type 2 diabetes.	Khetan *et al.*, 2018
SLDP	Years of education / Crohn’s disease	SLDP was applied to 46 diseases and complex traits together with 382 transcription factor binding annotations spanning 75 transcription factors and 84 cell lines predicted using ENCODE ChIP-seq data. Analyses yielded 77 significant annotation-trait associations, spanning six diseases and complex traits. Of note to the focus of this review, a positive association was found between years of education and genome-wide binding of BCL11A, which has also been identified in rare variant studies of intellectual disability. Less relevant to the review, but of equal importance, other associations detected included a positive association between IRF1 and Crohn’s disease.	Reshef *et al.*, 2018
s-LDSC	Alzheimer’s disease/ Parkinson’s disease	Alzheimer’s disease and Parkinson’s disease heritability were found to be enriched in regulatory annotations marking gene activity in immune cells.	Gagliano *et al.*, 2016
	Alzheimer’s disease / Bipolar disorder / schizophrenia	s-LDSC was applied across 48 disease and traits, using gene expression and chromatin data from a number of sources, including: ENCODE, GTEx, PsychENCODE, and the ImmGen project. Of note to the diseases mentioned throughout this review, a significant enrichment of heritability was found for Alzheimer’s disease, bipolar disorder and schizophrenia in myeloid cells, GABAergic (inhibitory) neurons and glutamatergic (excitatory neurons, respectively.	Finucane *et al.*, 2018
	Schizophrenia	Pyramidal cells, medium spiny neurons and certain interneurons were found to be implicated in schizophrenia, using both s-LDSC and MAGMA (another form of enrichment method).	Skene *et al.*, 2018
	Parkinson’s disease	Parkinson’s disease heritability was not found to enrich in investigated global and regional brain annotations or brain-related cell-type-specific annotations, but was found enriched in a curated lysosomal gene set.	Reynolds *et al.*, 2019
**Identifying genes of interest**			
coloc/fgwas	Subclinical atherosclerosis	cIMT and carotid plaque were used as measures of subclinical atherosclerosis. Using coloc, three genes were identified whose gene expression in both early and late advanced atherosclerotic arterial wall was associated with risk of atherosclerosis development. Notably, two of the genes identified associated with GWAS loci where GWAS association *P*-values did not meet the genome-wide significance threshold i.e. coloc analyses identified two additional risk loci, which would not have been identified otherwise. fgwas analyses found a high probability that the association of one locus with cIMT is driven by SNPs lying within open chromatin within adipose/derived mesenchymal stem cells, providing a potential downstream mechanistic explanation for this signal.	Franceschini *et al.*, 2018
coloc/TWAS	Parkinson’s disease	A combination of coloc and TWAS was used to identify 66 genes, whose expression or splicing in DLPFC and peripheral monocytes was significantly associated with Parkinson’s disease risk.	Li *et al.*, 2019
MR	Parkinson’s disease	Mendelian randomization was used to associate 14 genes associated with mitochondrial function also associate with Parkinson’s disease risk.	Billingsley *et al.*, 2019
moloc	Schizophrenia	moloc was used to identify 52 candidate genes associated with schizophrenia using eQTL and mQTL data derived from dorsolateral prefrontal cortex.	Giambartolomei *et al.*, 2018

ChIP = chromatin immunoprecipitation; cIMT = carotid artery intima thickness; DLPFC = dorsolateral prefrontal cortex.

Our focus is on tools that identify functional genomic annotations or genes of interest (Table 2), which are also available as online or downloadable software to be run on a local system. All tools we consider use association summary statistics rather than raw data because we note that several forces have driven the release of summary statistics over genotype-level data, including: protection of personal identifiable information; the rising trend of meta-analysis (with increasing data contributions from private companies such as 23andMe); the requirement for data release now imposed by many journals; and finally, large consortia, such as the Psychiatric Genomics Consortium (PGC), and the International Genomics of Alzheimer's Project (IGAP), endorsing the release of summary statistics. With the availability of summary statistics set to increase, use of summary statistics is becoming the reality for many GWAS analysts, which has and will continue to drive development of summary statistic-based tools.

**Table 2:** Tools to incorporate GWAS summary statistics with functional genomic annotations

Tool	Reference	URL	Input	Limitation(s)
**Identify annotations of interest**					
**Enrichment analysis of global trait-associated variants within annotations**					
fgwas	Pickrell, 2014	https://github.com/joepickrell/fgwas	Summary statistics, Annotation file	One ‘causal’ variant per locus
GARFIELD	Iotchkova *et al.*, 2019	https://www.ebi.ac.uk/birney-srv/GARFIELD/	Summary statistics, Annotation file, LD file, distance of each variant to the nearest transcription start site.	LD reference: provided LD information for UK10K, need recalculation for non-European studies
GoShifter	Trynka *et al.*, 2015	https://github.com/immunogenomics/goshifter	Variant map file, Annotation file, LD file	Critical to match on the number of variants in LD at each locus
GREGOR	Schmidt *et al.*, 2015	https://genome.sph.umich.edu/wiki/GREGOR	LD-pruned index variants, Annotation file, LD file	One ‘causal’ variant per locus
PAINTOR/fastPAINTOR	Kichaev *et al.*, 2014, 2017	https://github.com/gkichaev/PAINTOR_V3.0	Locus file; LD matrix file, Annotation matrix file	Assumes functional variants are shared at pleiotropic risk regions; If using multiple correlated GWAS traits, there is an assumption that no samples will overlap (often not the case)
**Enrichment of heritability of variants within annotations**					
SLDP^a^	Reshef *et al.*, 2018	https://github.com/yakirr/sldp	Summary statistics, Annotation file, LD Reference	Limited by the quality of annotations; LD reference to match ancestry of GWAS
s-LDSC^b^	Finucane *et al.*, 2015	https://github.com/bulik/ldsc/wiki/Partitioned-Heritability	Summary statistics, Annotation file, LD Reference	Restricted to common variants; Limited by the quality of annotations; LD reference to match ancestry of GWAS
SumHer	Speed and Balding, 2019	http://dougspeed.com/sumher/	Summary statistics, Annotation file, LD Reference	Limited by the quality of annotations; LD reference to match ancestry of GWAS
**Identify genes of interest**					
**Co-localization of eQTLs and trait-associated loci**					
coloc	Giambartolomei *et al.*, 2014	https://cran.r-project.org/web/packages/coloc/coloc.pdf	Summary statistics for GWAS and for eQTL	Restricted by quality of underlying eQTL data; One ‘causal’ variant per locus
eCaviar	Hormozdiari *et al.*, 2016	https://github.com/fhormoz/caviar	Summary statistics for GWAS and for eQTL, GWAS LD file, eQTL LD file	Restricted by quality of underlying eQTL data
enloc	Wen *et al.*, 2017	https://github.com/xqwen/integrative	Summary statistics for GWAS and for eQTL	Restricted by quality of underlying eQTL data
moloc	Giambartolomei *et al.*, 2018	https://github.com/clagiamba/moloc	Summary statistics for GWAS and QTL data.	Restricted by quality of underlying QTL data; One ‘causal’ variant per locus
**Estimate genetic association between disease risk and local gene expression**					
MRBase	Hemani *et al.*, 2018	http://www.mrbase.org	Choose input from existing database (or use R package)	Restricted by quality of underlying eQTL data; Mendelian randomization assumptions must be met
TWAS/ FUSION	Gusev *et al.*, 2016	http://twas-hub.org	Choose input from existing database	Restricted by quality of underlying eQTL data

Tools listed in alphabetical order.

a Signed linkage disequilibrium profile regression.

b Stratified LD score regression.

### Number of assumed ‘causal’ variants

Because of linkage disequilibrium (LD), correlations among nearby variants in the genome, there will be many genomic associations that are not ‘causal’. To assess enrichment of loci with functional genomic annotations, tools use LD to account for this non-independence, but they differ in the number of ‘causal’ variants they assume. fgwas and GREGOR, for instance, are examples of tools to assess enrichment of annotations; they assume one ‘causal’ variant per locus (Pickrell, 2014; Schmidt *et al.*, 2015). This assumption reflected GWAS at the time these tools were developed; that is, there was limited evidence to suggest there were multiple independent ‘causal’ variants at a single locus. However, as GWAS samples sizes have grown, and subsequently also our power to detect associations, it is becoming clear that this assumption may not stand true for some phenotypes where multiple ‘causal’ variants at a locus exist, such as the *SNCA* locus highlighted in Parkinson’s disease (Fritsche *et al.*, 2016; Campêlo and Silva, 2017). Thus, other tools have been developed for assessing enrichment in annotations, including GoShifter, PAINTOR and GARFIELD, which assume more than one ‘causal’ variant per locus (Kichaev *et al.*, 2014; Trynka *et al.*, 2015; Iotchkova *et al.*, 2019).

Co-localization between eQTLs and trait-associated variants—that is, assessing whether a variant affects both gene expression and trait-risk—can help identify genes of interest. As with tools for identifying annotations of interest, some assume one ‘causal’ variant per locus while others allow for multiple ‘causal’ variants. Until recently, there was limited evidence of secondary eQTL signals at one locus and so an assumption of one ‘causal’ variant in eQTL—GWAS co-localization was not a major issue. However, as sample sizes have grown, thus increasing statistical power, it has been demonstrated that secondary independent eQTL signals exist at some loci, violating this assumption (Dobbyn *et al.*, 2018). This violation can be circumvented using a series of ‘all-but-one’ conditional analyses, as performed by Dobbyn *et al.* that is, for each independent eQTL of a gene with multiple independent eQTL signals, an eQTL analysis would be performed including the effect of all other independent eQTLs. Alternatively, tools that allow for more than one variant can be used. Coloc and moloc, for example, are co-localization tools that assume one ‘causal’ variant per locus whereas eCaviar and enloc are tools that allow for multiple ‘causal’ variants (Giambartolomei *et al.*, 2014; Hormozdiari *et al.*, 2016; Wen *et al.*, 2017).

### Beyond genome-wide significant variants

To identify annotations of interest using GWAS summary statistics, one can assess whether genome-wide significant or sub-threshold loci of a complex trait are enriched within the annotation of interest, or using recently developed tools, one can assess whether the overall (genome-wide) heritability of a trait is enriched within an annotation of interest. This shift has been primarily driven by the emergence of stratified LD score regression (s-LDSC) and its variations (e.g. signed linkage disequilibrium profile regression, SLDP), which assess the contribution of an annotation to the overall heritability of a trait. Importantly, s-LDSC only requires association summary statistics and borrows LD information from reference data, in contrast to other methods for estimating heritability, which require genotype-level information (e.g. GCTA) (Finucane *et al.*, 2015; Reshef *et al.*, 2018). SumHer, a more recent development, also requires only summary statistics to estimate and then partition heritability for a group of variants that overlap with an annotation of interest, but allows the user to specify the heritability model; that is, heritability is allowed to vary throughout the genome, for instance according to minor allele frequency and local LD (Speed and Balding, 2019).

It is important to keep in mind that using association summary statistics as opposed to raw genotypes for estimating trait heritability, and thus also partitioning heritability, is still a relatively new endeavour; as such, these methods are not static, but rather are under continuous development. Furthermore, such methods rely on assumptions of the heritability model that may be well-suited to the genetic architecture of some traits but not others. Indeed, due to differences in the underlying models of heritability and approach to accounting for local LD, it has been shown that enrichment results can differ substantially depending on whether SumHer or s-LDSC was used (Gazal *et al.*, 2018; Speed and Balding, 2019). Finally, the tools in this domain are currently designed for common bi-allelic SNPs (>5% for s-LDSC and >1% for SumHer), and are not yet suitable for lower frequency variants or other forms of genetic variation such as small insertions or deletions, both of which are known to be important in schizophrenia (Kirov, 2015; Pardiñas *et al.*, 2018).

### Emergence of inferring unmeasured data

When using summary statistics, one class of information that needs to be inferred, as already alluded to, is the LD between the variants. Genotype data from reference panels, such as from the 1000 Genomes Project, is commonly used as a proxy to infer LD information not available from GWAS summary statistics. This strategy of inferring the LD structure based on LD information from reference data is likely to become unavoidable.

However, the inference of unmeasured data has expanded beyond LD, notably with methods developed to infer gene expression levels in the input dataset based on public expression data. To identify genes of interest for follow-up, transcriptome-wide association studies (TWAS), which test gene expression for association with a trait of interest, can be used (Gusev *et al.*, 2016). Many other tools exist for testing significant correlation between expression (which can be predicted using individual-level transcriptome reference data) and GWAS loci including, but not limited to, PrediXcan/MetaXcan and two-sample summary-data-based Mendelian randomization (SMR) (Gamazon *et al.*, 2015; Zhu *et al.*, 2016; Barbeira *et al.*, 2018).

### Ease of use

Regardless of whether the tool is for identifying annotations or genes of interest, the most widely used tools tend to have detailed instructions addressing installation, the format of the required input files, and the workflow either on a GitHub or wiki page. For instance, for tools that estimate the contribution of annotations to per-SNP heritability, s-LDSC is widely used; the primary s-LDSC paper (Finucane *et al.* 2015) has been cited almost 600 times as of June 2019. A likely factor contributing to its popularity is the ease of use; the s-LDSC-family of software is well documented and user-friendly with a good tutorial system in place. There is an active user discussion group, a wiki page with detailed instructions and examples, and all corresponding LD and genomic annotation files used in publications are available. Although much newer than s-LDSC, and thus not yet as widely used [the paper (Speed and Balding, 2019) has been cited 10 times as of June 2019], SumHer has good instructions, and a web version is in progress (SumHer Server). The server will be able to estimate heritability of a set of genomic annotations once a user uploads summary statistics in the required format, and could likely increase the ease of use for this tool as well. A built-in way to visualize the output of a tool also aids in utility. GARFIELD, for example, incorporates visualizations of its annotation enrichments results.

For identifying genes of interest, all of the co-localization tools discussed here are also well-documented on GitHub or the CRAN repository. Having options to run a tool as either a web-based application or on a local computer to meet the needs a wider range of users with differing computational skillsets or computational resources also influences ease of use. TWAS hub and MRbase, for example, are two well-maintained web interfaces with associated R packages that can be run locally (Gusev *et al.*, 2016; Hemani *et al.*, 2018). The results on the TWAS hub are powered by the software FUSION, a collection of tools that perform TWAS by integrating gene expression. The FUSION R package can be used, rather than the web interface, if users wish to conduct a TWAS locally. MRbase is primarily used to perform Mendelian randomization analyses to test for a causal relationship between the genetic contributions of one trait with another trait using association summary statistics, but can also assess the association between gene expression and genetic association summary statistics (Hemani *et al.*, 2018). MRbase can also be run locally as an R package instead of the web interface (Iotchkova *et al.*, 2019).

### Considerations when using tools to integrate annotations with GWAS summary statistics

From this overview of conceptual advances in computational tools that integrate functional genomic annotations with GWAS results it is important to highlight that all tools rely on assumptions, and thus have limitations (Table 2). However, as these assumptions evolve, so do the tools. For example, any of the tools discussed, rely on public sources of genome-wide data to compute and account for LD, and so as larger and more ethnically diverse reference panels become available to estimate LD, or as our understanding and the availability of genomic annotations that should be accounted for expands, this too must be reflected in the tools we use. The underlying assumptions will also change as our understanding of genetic architecture improves (such as discussed in terms of assuming one ‘causal’ variant per locus to assuming multiple variants).

Second, it is important to make explicit that all tools mentioned here rely on the quality and quantity of the input: the GWAS summary statistics and also the functional genomic annotations. For instance, co-localization of GWAS and eQTL signals heavily depend upon the integrity and accuracy of the eQTL data. Additionally, all tools discussed assume equal genome-wide coverage of the annotations, but we know from single cell RNA-sequencing that that is not yet necessarily a valid assumption.

### Summary

We discussed the influence of conceptual advances in the development of computational tools that integrate functional genomic annotations with GWAS summary statistics. As annotation quantity and resolution increase, one can imagine the exciting possibilities these data will have on refining existing or creating new tools to pinpoint variants or genes of interest and biological pathways in the relevant cell type(s). For instance, as sample sizes increase for expression studies, statistical power can be boosted to detect eQTLs of low effect in previously unidentified loci or potentially multiple independent ‘causal’ signals at known eQTL loci. As the brain-related genomic annotations at increasing resolution become more prominent, their integration into tools has huge promise to increase our understanding of the biology of neurodegenerative and neuropsychiatric disorders.

## To the future

### Extending current axes of information

#### Cellular resolution

Increasingly, we are having to reconsider our conceptual understanding of cell type and this will have implications for the generation of functional annotations. A recent single-cell RNA-sequencing study of the mouse hippocampal CA1 area suggested that modelling of cell types requires continuous modes of variation in addition to discrete cell classes; that is, some cell classes exist on a common genetic continuum (Harris *et al.*, 2018). Inherent within this spectrum is cellular state, which reflects the physiological condition of a given cell, whether it be the degree of differentiation or activation in response to a stimulus. To study such dynamic processes ‘pseudotime analysis’, also known as trajectory analysis, has emerged, which allows users to order cells along a deterministic or probabilistic trajectory based on similarities in their gene expression profiles (Rostom *et al.*, 2017; Packer and Trapnell, 2018). One of the limitations of this analysis has been the overwhelming number of methods available, with limited assessment of performance and reproducibility across datasets; however, with large-scale benchmarking efforts underway (Saelens *et al.*, 2019), it is likely that robust annotations addressing the dynamics of cellular state will soon emerge. We predict that these annotations will greatly contribute to our understanding of complex neurological disorders, given that risk loci can arguably regulate transitions from one cellular state to another (e.g. during neurodevelopment in the case of neuropsychiatric disease or in response to stimulus in the case of neurodegenerative disease).

#### Molecular resolution

Brain-related expression data have been principally evaluated at two levels of transcriptomic organization: gene and exon. However, with recent consortia-led data releases, these levels have been complemented with evaluation of exon-exon junctions and gene isoforms (Collado-Torres *et al.*, 2018; Gandal *et al.*, 2018; PsychENCODE Consortium, 2018). The significance of isoform-level data at tissue-level resolution is emphasized by estimates that 92–94% of human genes undergo alternative splicing and by the tissue-specificity of alternative splicing events in humans (which for some organs, including the brain, is conserved across mammals) (Yeo *et al.*, 2004; Wang *et al.*, 2008; Merkin *et al.*, 2012; GTEx Consortium, 2015; Melé *et al.*, 2015; Li *et al.*, 2018). With respect to the utility of isoform-level data in disease, a study of transcriptome dysregulation in three neuropsychiatric disorders found that isoform-level changes, as opposed to gene-level changes, demonstrated the largest effect sizes in diseased brains, were most reflective of genetic risk and provided greatest disease specificity in differential expression analyses and construction of co-expression networks (Gandal *et al.*, 2018). Although, our knowledge of isoform specificity to biological pathways remains incomplete (most gene set enrichment analyses are currently run at gene level) and this currently limits the utility of capturing this information, this is expected to change as the quantity of isoform-level data increases (such as through the emergence of longer-read sequencing techniques or the growing throughput of full-length single-cell transcriptomic assays). Thus, we expect isoform-level data to emerge as an important component within the axis of molecular resolution.

### New axes of information

While single-cell epigenomic and transcriptomic techniques enable molecular cell-type classification, it is important that these molecularly-defined cell types are related back to their morphological, electrophysiological and spatial phenotypes. This goal is possible using techniques, such as Patch-seq, that combine whole-cell electrophysiological patch-clamp recordings, single-cell RNA-sequencing and morphological characterization, albeit in a very labour-intensive and low-throughput manner (Cadwell *et al.*, 2016, 2017; Fuzik *et al.*, 2016). Likewise, methods are emerging in the field of spatial transcriptomics—such as multiplexed error robust fluorescent *in situ* hybridization (MERFISH)—that provide opportunities to interrogate spatiotemporal regulation of the brain transcriptome. With time, such analyses could imaginably be extended to measurement of spatiotemporal regulation during different mental activities or in disease, further increasing the number of available axes of information and extending the diversity of functional genomic annotations available for integration with GWASs (Crosetto *et al.*, 2015; Lein *et al.*, 2017).

### Integrating across axes of information

As the number of functional genomic annotations from the brain increases, the opportunity for the multi-omic combination of many layers of information, such as through the use of machine-learning techniques, becomes feasible (Packer and Trapnell, 2018; Zitnik *et al.*, 2019). Indeed, recent work by the PsychENCODE Consortium highlights the potential of combining multiple layers of information in an interpretable deep-learning model, an approach that both identified key pathways associated with disease and was shown to improve disease prediction compared to polygenic risk scoring and traditional additive models (Wang *et al.*, 2018). Many data-trained classifiers making use of hundreds or thousands of genomic annotations have been developed, including DANN, GWAVA, SilVA, CADD, deltaSVM and DeepSEA (Buske *et al.*, 2013; Kircher *et al.*, 2014; Ritchie *et al.*, 2014; Lee *et al.*, 2015; Quang *et al.*, 2015; Zhou and Troyanskaya, 2015). In addition to identifying genetic variants of interest, these methods have the potential to infer the importance of each annotation (keeping in mind collinearity of annotations, and feature selection biases) and to identify information common to all annotations. This work sets the seeds for future machine learning algorithms, which will need to be scaled up to match the quantity and size of emerging annotations, particularly in the field of single-cell analysis.

### Predicting across axes of information

With its ability to find patterns and structure large amounts of data that are otherwise too complex for the human brain, machine learning is also aptly positioned to predict one molecular phenotype based on other molecular phenotypes. Whether this is predicting differential chromatin accessibility from transcriptomic data or vice versa (Lake *et al.*, 2018), predicting transcription factor binding from chromatin accessibility and gene expression (Keilwagen *et al.*, 2018), or predicting splice sites from pre-mRNA (Jaganathan *et al.*, 2019), this ability not only enables prediction of molecular phenotypes we cannot assay, but also allows imputation of missing values across molecular phenotypes that have been assayed in parallel. With the emergence of technologies like single-cell multi-omics (simultaneous assaying of multiple molecular phenotypes, which enables generation of models relating epigenomic variation and transcriptomic dynamics) that are plagued by sparseness of data, we imagine that there will be a surge in the number of available predictive tools (Macaulay *et al.*, 2017; Zitnik *et al.*, 2019).

## Conclusion

The past few years have seen a growth in the number of brain-relevant functional genomic annotations with increasingly high cellular and molecular resolution, a trend that is set to continue across current and new axes of information. Likewise, there has been an increase in the number of summary statistic-based computational tools for integrating these annotations with genetic association results to identify the genes, pathways and cell types relevant to disease. This growth comes at an opportune time when GWASs are growing ever larger, thus improving the power for such biological interpretation. Given the amount of data emerging, it will be important in the coming years to ensure that tools also scale accordingly, with machine learning approaches likely to become central players. Furthermore, to ensure efforts remain focused, it will be crucial to continually assess the value of new annotations to GWAS interpretation, a task that necessitates a more dynamic and immediate flow of information between annotation generators and their users than what is currently the norm. Given the ability of consortia to drive efforts within the fields of GWAS and annotation generation, and the increasing trend toward team science collaborative efforts, we do not foresee that this will be an issue and predict that this will produce a substantial increase in our understanding of human brain diseases.

## Abbreviations

eQTL =: expression quantitative trait loci
FACS =: fluorescence-activated cell sorting
GWAS =: genome-wide association study
LD =: linkage disequilibrium
s-LDSC =: stratified LD score regression
TRAP =: translating ribosome affinity purification
